# Supplementary material for: The interactome of intact mitochondria by cross-linking mass spectrometry provides evidence for coexisting respiratory supercomplexes
Source: Mol Cell Proteomics. 2017 Dec 8;17(2):216–32. doi: 10.1074/mcp.RA117.000470 (PMC5795388; doi:10.1074/mcp.RA117.000470)
Supplement: Supplemental Data [file supp_RA117.000470_133922_0_supp_23978_fzffwf.zip › spectra_annotation/mito_DR_spectra_annotation/246-1-3-1-3-1.pdf]

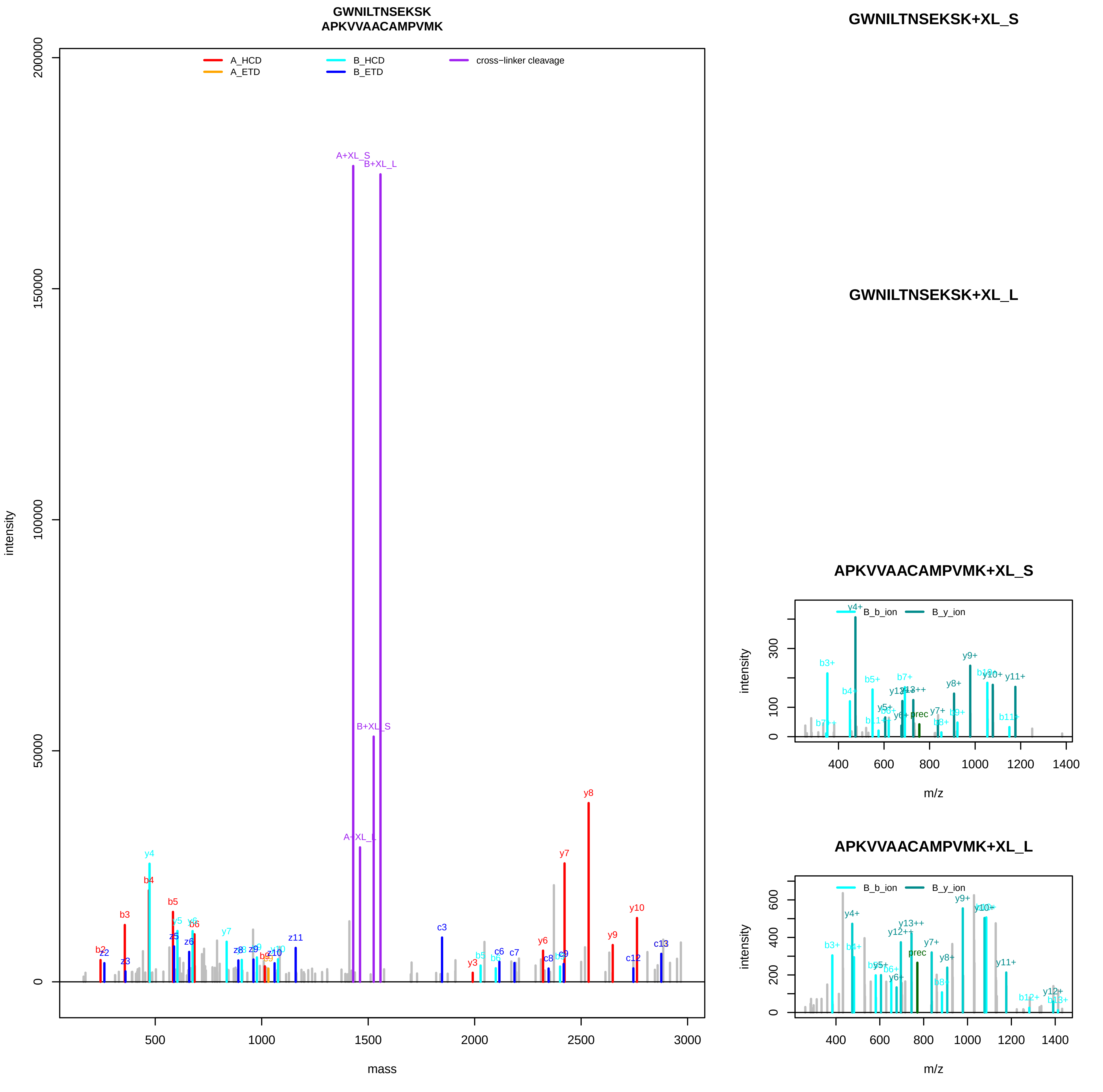
GWNILTNSEKSK
APKVVAACAMPVMK
A_HCD
A_ETD
B_HCD
B_ETD
cross−linker cleavage
A+XL_S
B+XL_L
B+XL_S
A+XL_L
intensity
mass
200000
150000
100000
50000
0
500
1000
1500
2000
2500
3000
y8
y7
y10
y9
y6
y3
b2
b3
b4
b5
b6
y4
y5
y7
z2
z3
z5
z6
z8
z9
z10
z11
c3
c6
c7
c8
c9
c12
c13
GWNILTNSEKSK+XL_S
GWNILTNSEKSK+XL_L
APKVVAACAMPVMK+XL_S
B_b_ion
B_y_ion
intensity
m/z
0
100
300
400
600
800
1000
1200
1400
y4+
b3+
b5+
b7+
y9+
y10+
y11+
y8+
prec
APKVVAACAMPVMK+XL_L
B_b_ion
B_y_ion
intensity
m/z
0
200
400
600
y9+
y10+
y13++
y12++
y7+
y8+
y11+
b3+
b4+
y6+
prec
b12+
y12+
b13+
